# Cauda Equina in Pregnancy: Early Management and Outcome

**DOI:** 10.7759/cureus.82274

**Published:** 2025-04-14

**Authors:** Dara Yildiz, Shane C Irwin, Gul Helin Ozgokce, Conor Ledingham, Bridget Hughes

**Affiliations:** 1 Trauma and Orthopaedics, Mayo University Hospital, Castlebar, IRL

**Keywords:** antenatal surgical care, cauda equina syndrome (ces), disc extrusion in pregnancy, lumbar disc herniation, multidisciplinary surgical approach, pregnancy-related spinal pathology, spinal decompression surgery, surgical timing in ces

## Abstract

Cauda equina syndrome (CES) is a rare but serious clinical presentation characterised by compression of the lumbosacral nerve roots below the conus medullaris, resulting in lower back pain, bilateral sciatica, motor and sensory deficits, and bladder or bowel dysfunction. Its occurrence in pregnancy is exceptionally rare and poses significant diagnostic and management challenges.

We present the case of a 34-year-old woman at 22 weeks’ gestation following in vitro fertilization (IVF) who developed acute bilateral leg pain, lower limb weakness, and urinary retention following a three-day history of backache. Neurological examination revealed reduced lower limb power, saddle anaesthesia, and loss of perianal sensation with absent ankle reflexes. MRI demonstrated a large L5/S1 disc extrusion completely filling the spinal canal.

Following multidisciplinary assessment, she underwent urgent surgical decompression via left L5/S1 laminotomy within eight hours of symptom onset. A large sequestrated disc fragment was removed, and the nerve root canal decompressed. Post-operatively, the patient reported immediate relief of radicular pain and gradual return of bladder function and motor strength.

Her pregnancy continued without complication, and she delivered a healthy baby by elective caesarean section at 38 weeks. At four-month follow-up, she had made a full recovery with complete resolution of neurological symptoms apart from mild residual back pain.

This case highlights the importance of prompt recognition and early intervention in CES during pregnancy. A multidisciplinary approach is essential to facilitate timely imaging and surgery while ensuring maternal and fetal safety.

## Introduction

Cauda equina syndrome (CES) is a rare clinical presentation characterised by a pattern of neuromuscular and urogenital symptoms as a result of compression of multiple lumbosacral nerve roots below the level of the conus medullaris. These symptoms include lower back pain, sciatica which can be unilateral or bilateral, saddle sensory disturbances, bladder and bowel dysfunction, and variable lower extremity motor and sensory loss. It is most commonly caused by massive disc herniation but other pathologies such as spinal lesions and tumours, haematoma and trauma can be implicated.

In the general population it is a rare condition [1] estimated at 1-3 per 100,000 patients and 1-2% of patients undergoing lumbar disc surgery. In pregnancy, it is even rarer, with a small number of cases cited in the literature [2-4].

Urgent surgical decompression is recommended in all cases but in pregnant women special considerations are required regarding anaesthesia, obstetric care as well as surgical positioning and a multidisciplinary approach is vital [5].

## Case presentation

A 34-year-old female presented with a history of an in vitro fertilization (IVF) pregnancy of 22 weeks’ gestation complaining of acute lower backache for three days and a six-hour history of pain radiating down the back of both legs, worse on the left side. She complained of weakness in both legs and difficulty urinating.

Her pregnancy up until then had proceeded without any complications. Clinical examination revealed reduced power 4/5 in myotomes of L4, L5 and S1. There was decreased sensation in dermatomes L5, S1, S2, and S3. PR examination revealed decreased perianal sensation and reduced anal tone. Ankle reflexes were absent bilaterally.

**Figure 1:**
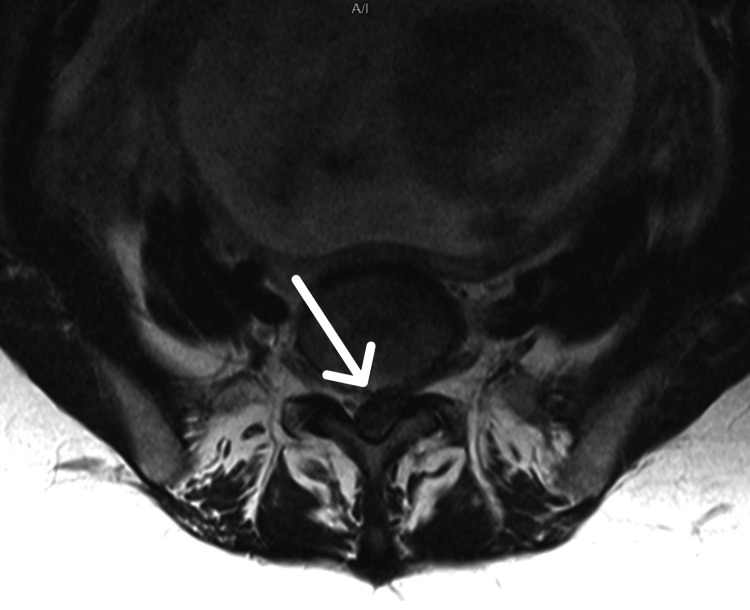
MRI Lumbar Spine: T2-weighted axial view at L5/S1 level demonstrating almost complete filling of the spinal canal with extruded disc

**Figure 2:**
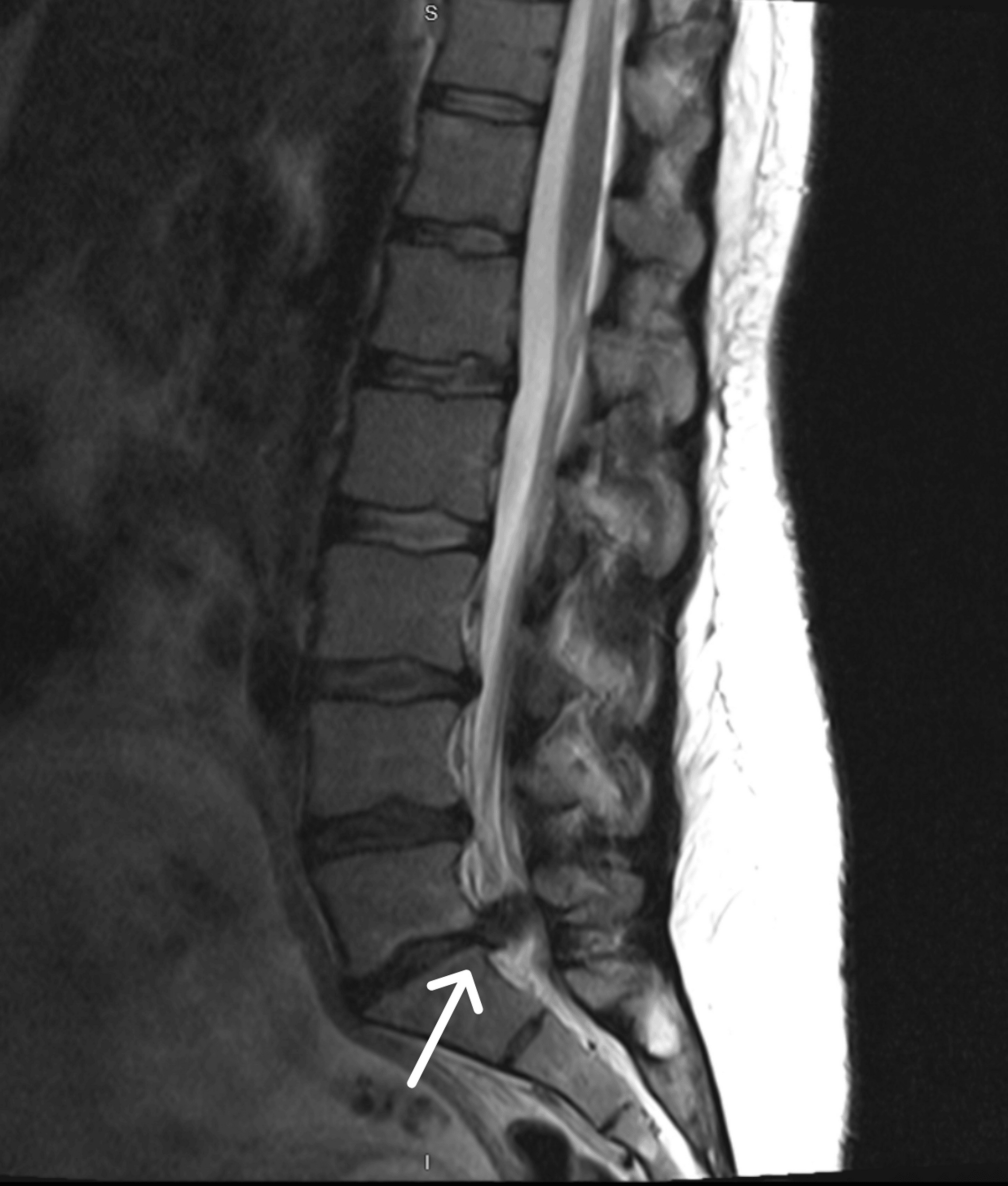
MRI Lumbar Spine: T2-weighted sagittal view demonstrating large L5/S1 disc protrusion/extrusion

A urinary catheter was inserted with a residual volume of 1000 ml. An MRI scan was performed with sagittal and axial images showing a large disc protrusion/extrusion at the L5/S1 level, completely filling the spinal canal (Figure 1, Figure 2).

The patient was evaluated from an obstetric and anaesthetic point of view and, after informed consent was obtained, was taken to theatre for urgent decompression. MRI scan and transfer to theatre occurred within two hours of presentation, eight hours after onset of CES symptoms.

The patient was positioned prone on a Montreal mattress with the abdomen placed in the hollow in order to avoid excessive pressure on the abdomen and uterus. A left paraspinal approach was used and a single intraoperative X-ray taken with the image intensifier in order to confirm the level.

**Figure 3:**
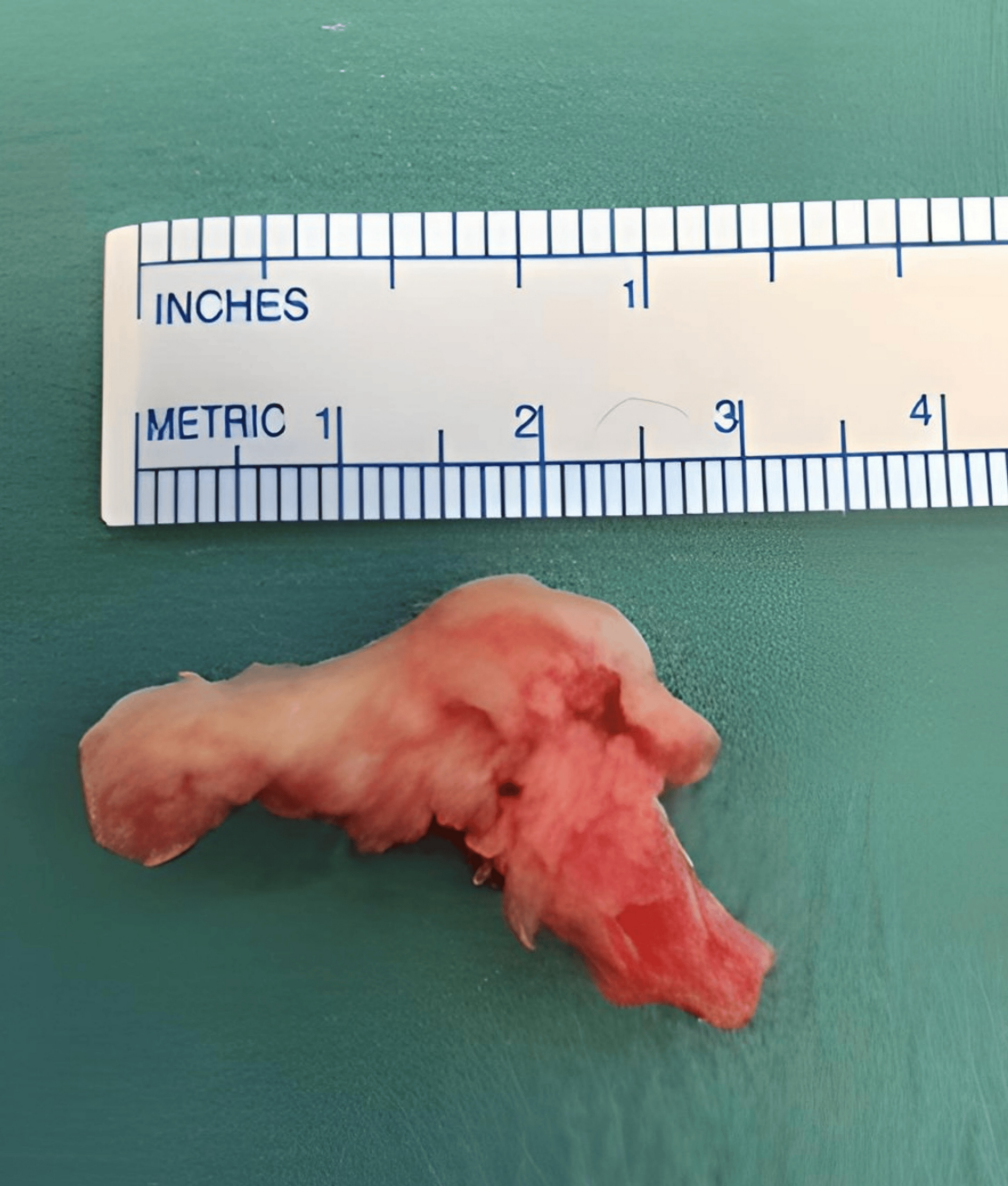
Large extruded disc fragment

A left L5/S1 laminotomy was performed. The S1 nerve root was retracted and a large sequestrated disc was identified and excised (Figure 3).

The disc space was cleared of any remaining disc and the nerve canal decompressed. A large volume of disc material was obtained (approximately 3.1 cm).

Symptomatic improvement was reported immediately after surgery, with leg pains much improved. Urinary catheterisation was continued for two weeks post-operatively and sluggish bowel movements were managed with simple laxatives. T.E.D. anti-embolism stockings and low molecular weight heparin were administered in the post-operative period to reduce the risk of thromboembolic events.

Lower limb weakness improved and was reported as normal after one week. Saddle anaesthesia and bladder sensation improved within two weeks, and complete bladder voiding was monitored with bladder ultrasound.

The remainder of the pregnancy proceeded uneventfully, and a healthy baby was delivered by elective caesarean section at 38 weeks’ gestation. Clinical review at four months post-operatively revealed a complete resolution of symptoms apart from some mild back pain, with full recovery of bladder and bowel symptoms.

## Discussion

The diagnosis of CES in pregnancy is difficult given the frequency of low back symptoms in pregnancy and the rarity of the condition. Patients often delay seeking medical attention and junior medical staff frequently do not recognise the ‘red flag’ signs and symptoms, which can result in delays in obtaining MRI scanning and time to surgery. Whilst red flag guidelines may vary between different countries [6], all encompass those of bladder dysfunction, bowel dysfunction, pain and/or altered sensation in the legs, loss of sexual sensation, and saddle numbness.

MRI scanning is the investigation of choice and exposure to serial echo planar MRI in utero has demonstrated no adverse effects to the growing foetus [7]. Surgical decompression is recommended at any stage in pregnancy as the optimal treatment [8,9].

It is generally recognised that time to surgery is critical for a good outcome and patients need to be counselled with regard to the possibility of persistent neurological symptoms, particularly bladder and bowel dysfunction, in cases of delayed presentation. A meta-analysis by Ahn et al. involving 322 patients from 42 publications indicated that there was a significant advantage to treating patients within 48 versus more than 48 hours after the onset of CES. A significant improvement in sensory and motor deficits as well as urinary and rectal function occurred in patients who underwent decompression within 48 versus after 48 hours. They also stated, however, that no significant improvement in surgical outcome was identified with interventions less than 24 hours from the onset of CES compared with patients treated within 24-48 hours [10].

Kohles et al. critically reassessed the Ahn paper and concluded that although an advantage existed in treating patients within 48 hours, the earlier the surgery - including within the 24-hour period - the better the outcome [11]. Barker et al. looked at the long-term outcomes of CES in a group of patients who had lumbar decompression surgery and noted that persistent severe back pain and ongoing autonomic dysfunction were frequently reported at a mean follow-up of five years [12]. Hazelwood et al. noted similar findings with a high prevalence of long-term bladder, sexual and physical dysfunction in CES patients following decompression surgery that had presented with urinary retention [13].

## Conclusions

In this case, early diagnosis and decompression within eight hours of the onset of symptoms resulted in a favourable outcome with no long-term sequelae. Surgery for CES in pregnancy presents unique challenges that require a timely and multidisciplinary approach to optimise outcome for the patient and the safe continuation of pregnancy to full term.
